# The time window before biological AI spreads: To prevent the misuse of biological AI models with dual-use capabilities requires governance of the embargo window

**DOI:** 10.1038/s44319-026-00886-2

**Published:** 2026-07-17

**Authors:** Benjamin D Trump, Cindy Groff-Vindman, Christopher L Cummings

**Affiliations:** 1 North Carolina State University, Raleigh, NC USA; 2 CINBIO LLC, Dumfries, VA USA; 3 U.S. Army Engineer Research and Development Center, Raleigh, NC USA

**Keywords:** Computational Biology, Economics, Law & Politics, Science Policy & Publishing

## Abstract

Frontier AI model embargo is emerging as a mechanism for shaping governance for biological AI. We explain how a pre-deployment embargo can create the time and structure needed for meaningful oversight, but only if it is grounded in clear capability-based evaluation, informed by independent multidisciplinary expert analysis, and designed to identify unique hazards before public release.

**Figure:**
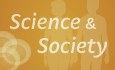

Frontier artificial intelligence is shortening the time between biological insight and practical use. A model, dataset, or workflow can reveal a capability of concern after development has begun, while there is still a short chance to act before broad diffusion. That chance is no longer theoretical. Anthropic held the Claude Mythos Preview outside general release while selected defenders used it to find software vulnerabilities. A June 2026 White House Executive Order created a voluntary pre-release access channel for covered frontier models in cybersecurity. OpenAI then extended trusted access into life sciences through Rosalind Biodefense and a biodefense action plan. These examples point to the same fragile period: the embargo window, when a developer knows enough to worry, and outside institutions still have time to strengthen defenses. For biological AI, that window needs clear capability triggers, secure access for auditors and public-interest defenders, concrete defensive work, and public reporting of methods and aggregate findings. It can buy time for preparedness. It will only deserve trust if it stays narrow, accountable, and open to the institutions that will carry the risk.

For much of modern biotechnology, biosecurity and biosafety oversight to prevent the nefarious use of biological research, tools, and agents has depended on objects that institutions could see. A funder could review a proposal. A biosafety committee could examine a protocol. A journal could halt the publication of a manuscript. Export-control officers could inspect shipments. Synthesis providers could screen orders. These control gates are not always perfect and sometimes slow, but they have been efficient as they sit at clearly identifiable bottlenecks in the research pipeline.

For much of modern biotechnology, biosecurity and biosafety oversight to prevent the nefarious use of biological research, tools and agents has depended on objects that institutions could see.

Biological AI challenges this system of controls. A potential dual-use capability may sit in model weights, a private dataset, a plug-in, or an agentic workflow that joins literature search, sequence interpretation, coding, procurement advice, or experimental planning. The same system may support drug discovery or outbreak response on Monday and raise a biosecurity concern by Friday. By the time conventional oversight begins to analyze its dual-use capability, the workflow may have already been adopted by other research groups.

The same system may support drug discovery or outbreak response on Monday and raise a biosecurity concern by Friday.

Collingridge’s dilemma captures this timing problem: early intervention has steering power but relies on insufficient information, while late intervention has sufficient evidence but less control (Collingridge, 1980). Frontier biological AI compresses the time between those points. The familiar sequence of build, publish, diffuse, and regulate leaves too little room for deliberation when a capability can move through scientific collaborations before most institutions become aware of its damaging potential.

The practical question is simple. What happens after an AI capability becomes visible to a developer or evaluator and before it becomes widely available? That period already exists. Companies use it when they run private previews. Governments use it when they request pre-release access. Public-health institutions will need it when the next model can speed legitimate response and lower barriers to misuse at the same time. We call that period the embargo window.

## Glasswing made the window visible

Project Glasswing is an instructive example, even though it began in cybersecurity. Anthropic developed the Mythos AI model to specifically find vulnerabilities in software. The company initially released the model to roughly 50 partners for discovering and fixing defensive vulnerabilities and stated that those partners had found more than 10,000 high- or critical-severity flaws. Anthropic then expanded the program to about 150 additional organizations in more than 15 countries.

Those numbers point to a sobering fact. According to Anthropic’s red-team assessment, Mythos Preview identified vulnerabilities across major operating systems and browsers, even though responsible disclosure kept many findings out of public view. At that scale, discovery can run faster than institutions’ abilities to validate and repair vulnerabilities.

For biology, there is a practical lesson. A pause or an embargo matters only if it can be used to fix problems during it. Glasswing did more than hold back a model. It enabled experts to reduce danger before wider release. In software, that work means vulnerability triage and patching. In biology, it could mean synthesis-screening updates, functional-prediction review, diagnostic validation, countermeasure prioritization, biosurveillance workflows, procurement safeguards, and public-health exercises.

A pause or an embargo matters only if it can be used to fix problems during it.

There is a cautionary tale too. Glasswing relied on selected partners with large security teams, trusted relationships, and close alignment with the developer. Biology is less centralized. Relevant expertise is scattered across universities, hospitals, public-health laboratories, synthesis providers, clinical manufacturing, epidemiology, and international surveillance networks. A biological embargo window limited to a small circle of firms and national partners would miss much of the expertise needed to evaluate frontier models. It would also start to look like a scientific hierarchy.

Cybersecurity already has a vocabulary for the next step: vulnerability triage, responsible disclosure, coordinated patching, and public advisories. Biology has fragments of equivalent practices, but no settled protocol for dealing with a model-discovered biological concern that can involve many partners: developers, public-health laboratories, synthesis providers, funders, or publishers. A biological embargo window could supply that protocol. It would name the evidence, the people who need early access, and the duties that come with access before broad diffusion.

## The executive order changed the politics

The White House Executive Order of June 2, 2026, changed the status of the pause. Its subject is cybersecurity, yet biology cannot ignore it. It directs federal agencies to develop a classified benchmarking process through which developers may voluntarily give the federal government access for up to 30 days before planned release (White House, 2026). Biology should draw two lessons from the order. One, that the embargo period before broader release is now a legitimate point for public action. Second, a voluntary and partly classified channel may not be sufficient for biological risk assessment. Thirty days is a useful embargo time for a software release. It is a poor proxy for biological readiness.

In biology, an embargo window should close against an audit plan, a screening update, a diagnostic validation package, a red-team summary, a public-health exercise, or a documented decision that release can proceed. Scientific trust still requires public trigger categories, conflict-of-interest rules, independent audit requirements, and aggregate reporting about what was evaluated and what changed during the embargo.

## OpenAI brought the question home

The biological version of Mythos is now reality. OpenAI announced Rosalind Biodefense to trusted developers and government partners working on biodefense, public health and pandemic preparedness and published a biodefense action plan. It describes Government Trusted Access for frontier life-science capabilities, with bounded mission work, authorized users, data protection and expert review. It also commits to sharing high-level findings and methods when disclosure can strengthen governance without creating information hazards. With Rosalind Biodefense, trusted access in biology has moved from an abstract policy proposal into a concrete action plan.

With Rosalind Biodefense, trusted access in biology has moved from an abstract policy proposal into a concrete action plan.

That move raises an unavoidable question: who counts as a trusted defender? A narrow answer would reproduce existing privilege through cloud contracts, government relationships, and institutional prestige. A defensible answer would start with the people who can reduce risk in practice: synthesis-screening teams, diagnostic laboratories, biosafety officers, agricultural-health authorities, clinical researchers, public-health agencies, and scientists in regions likely to bear outbreak consequences. Trust should rest on role, competence, accountability, and safeguards.

The public record should show which defensive tasks improved, which risks appeared, who checked the results, and why access terms were changed or kept in place. An independent audit is central here. AVERI defines frontier AI auditing as third-party verification of developers’ safety and security claims, based on deep, secure access to non-public systems and practices. The same logic applies to biological AI.

Independent evaluation of the capabilities of concern supplies the other party. Drawing on decades of dual-use review in the life sciences, Pannu and colleagues argue that biological AI evaluations should prioritize capabilities that could enable high-consequence harms and that such risks should be evaluated before deployment (Pannu et al, 2025). The ability of AI to analyze, compare, and design biological ‘code’ creates hazards that differ from those posed by conventional software because it can accelerate scientific discovery and reveal previously unrecognized biological insights. Accordingly, evaluations should also prioritize AI systems capable of generating novel biological insights or biotechnical discoveries with real-world implications. That helps define what to evaluate. The embargo window defines when public-interest defenders can evaluate.

## Evo 2 made the data problem visible

The capabilities of a model also depend on the data from which it has learned. Evo 2 was trained on approximately 9 trillion DNA base pairs from a curated genomic atlas spanning all domains of life (Brixi et al, 2026). Through next-nucleotide prediction across this diverse dataset, the model learned patterns shaped by natural selection and can predict functional impacts of genetic variation.

That result matters for governance, not just performance. Evo 2 shows that biological foundation models are beginning to derive biological fluency directly from massive sequence data. Groff-Vindman and colleagues describe this as part of the wider convergence of AI and synthetic biology, in which models increasingly support automated design, build, test, and learn cycles (Groff-Vindman et al, 2025). The governance challenge now includes what AI systems can infer from data at scales that ordinary review cannot inspect.

Brixi et al (2026) also report the deliberate exclusion of viruses capable of infecting human cells from the Evo 2 training dataset. Removing those sequences weakened both language-model performance and downstream prediction on human viruses. Dataset composition therefore shapes capability. Proprietary, state-controlled or otherwise inaccessible datasets can create capability uncertainty that outsiders cannot easily evaluate.

Embargo-period evaluations therefore need to account for plausible capability gains from additional biological data, in addition to demonstrated capability at one point in time. The security value of biological sequence data may extend beyond sequences from regulated pathogens or toxins. Large collections of genomic data, even from unregulated organisms, may contribute to advanced biological capability when aggregated for model training. A recent National Academies assessment similarly treats biological datasets for AI training as a source of biosecurity concern (National Academies of Sciences, Engineering, and Medicine, 2025).

## Biology needs its time

Cybersecurity offers useful strategies for responsible disclosure, red-teaming, and patching. But while software vulnerabilities can often be analyzed and patched on compressed schedules, biological risk assessment for model behavior may take weeks or months. Risk concerns may lie well short of pathogen construction. Improved sequence-to-function inference, design of difficult-to-screen variants, assistance with experimental troubleshooting, procurement guidance, or orchestration across databases and tools can each matter in the right context. The riskiest future systems may simply link ordinary-looking steps: search, design, simulate, troubleshoot, and document. Traditional oversight has often examined pieces of that chain. Biological AI requires review of the whole chain.

… while software vulnerabilities can often be analyzed and patched on compressed schedules, biological risk assessment for model behavior may take weeks or months.

DNA synthesis screening offers a concrete starting point because it already sits near a material choke point. Wheeler et al (2024) have argued for a common global baseline for screening. Wittmann et al, 2025 showed that generative protein-design tools can stress screening, and that defensive tools can be strengthened when evaluators receive an early warning signal and a usable testbed. Early access could work when it is able to improve a safeguard before risky outputs become routine. Other biological defenses will need their own tests. A model that improves the interpretation of genomic or environmental signals has to be tested against public-health workflows, false-alarm costs, and response capacity. A tool that assists experimental planning has to be reviewed for how it handles biosafety protocols, procurement, and protocol misuse. An embargo only matters if defensive systems can absorb the model’s risks.

## What makes the window defensible

An embargo window’s legitimacy rests on clear definitions. The trigger for an embargo should reflect the tested capability. A model family, brand name, or company press release is too blunt. The trigger should name the function that creates concern, such as a demonstrated ability to lower barriers in a sensitive workflow, bypass a safeguard, or combine biological reasoning with agentic execution in ways existing reviews did not anticipate. Once the function is named, restrictions can be narrower and easier to lift.

Similarly, it requires clear exit criteria to close the window. A useful window opens with a written account of the trigger, the evidence needed for release, the evaluator groups invited, and the authority empowered to decide whether the criteria have been met. For biology, the endpoint may be a completed audit, an updated screening protocol, a validated diagnostic workflow, a revised access policy, or a public explanation of residual risk. Closure should be observable.

Access is the hardest choice. A credible window needs public-health agencies, synthesis-screening experts, biosafety professionals, independent auditors, relevant academic groups, and qualified institutions outside the developer’s home jurisdiction. Managed access can protect sensitive capabilities while allowing participation of external reviewers. Careful access is more defensible than narrow access. That access has to be legible to outsiders. Trigger categories, benchmarks, audit procedures, conflict-of-interest rules, results, and release criteria should be published in forms that avoid misuse. Without that layer, outsiders cannot tell whether the window produced preparedness or merely protected a developer’s interests.

The World Health Organization’s guidance on responsible use of the life sciences, synthesis-screening standards, and emerging AI-safety institutes offer partial directions for managing an embargo window (Wheeler et al, 2024; Trump et al, 2026). Used together, they can help separate genuine defensive urgency from ad hoc gatekeeping. International participation is important because biological risks cannot be contained within borders.

## Pilot before crisis

The next step is to pilot the window on defined problems. One pilot could test how a model stresses nucleic-acid screening and how screening providers could update their tools in response. A second could examine whether it improves diagnostic or surveillance workflows under realistic false-positive and false-negative costs. A third pilot could test countermeasure workflows. Each pilot should produce public methods, an audit summary, and a record of defensive changes made before wider access.

Federal coordination has a starting point in the White House order, but biology will need a wider variety of institutions and expertise. Biological AI evaluation has to draw on public health, biosafety, clinical medicine, agriculture, synthesis screening, laboratory operations, and international governance. A US-only voluntary channel would be too narrow for assessing biological risks, as outbreaks, supply chains, and scientific collaboration are global. Waiting for a perfect multilateral treaty would leave the window open to company discretion in the meantime. Instead, the way forward would be to pilot first, publish methods, widen participation, and let the evidence guide formalization.

Biological AI evaluation has to draw on public health, biosafety, clinical medicine, agriculture, synthesis screening, laboratory operations and international governance.

Recent events changed the argument. Glasswing showed that a developer can hold back broad release to give defenders a head start. The Executive Order made pre-release access part of federal security policy. OpenAI’s life-science program showed that trusted access is now an active deployment model for biological AI. Evaluation of capabilities of concern and an independent audit offer ways to discipline the pause. The parts are on the table. Emergency should not be the default plan.

Scientists should insist on a simple exchange. During the embargo window, developers may restrict sensitive capabilities. In return, they should provide enough evidence for independent auditors, public-interest defenders, and affected institutions to judge whether the restriction serves preparedness. The bargain is temporary secrecy for demonstrable public work, followed by a public account of what changed.

A pause before release can be misused. It can become a moat, a liability shield, or a way to move consequential decisions out of public view. It can also protect open science by giving institutions time to strengthen safeguards before the most sensitive capabilities spread. The embargo window is already forming. Biology should decide now whether it becomes a narrow tool for preparedness or a private gatekeeping system dressed in the language of safety.

## Supplementary information

Peer Review File
