# Supplementary material for: The time window before biological AI spreads: To prevent the misuse of biological AI models with dual-use capabilities requires governance of the embargo window
Source: EMBO Rep. 2026 Jul 17;27(15):4204–7. doi: 10.1038/s44319-026-00886-2 (PMC13457865; doi:10.1038/s44319-026-00886-2)
Supplement: Supplementary file 1 [file 44319_2026_886_MOESM1_ESM.pdf]

EMBO reports

The time window before biological AI spreads

Benjamin Trump, Cindy Groff-Vindman, and Christopher Cummings

Corresponding author(s): Benjamin Trump (Bdtrump@umich.edu)

Review Timeline:

Submission Date:	12th Jun 26
Accepted:	13th Jul 26

Editor: Holger Breithaupt

Transaction Report: This article was editorially reviewed.
